# The immunogenetic impact of European colonization in the Americas

**DOI:** 10.3389/fgene.2022.918227

**Published:** 2022-08-05

**Authors:** Evelyn Jane Collen, Angad Singh Johar, João C. Teixeira, Bastien Llamas

**Affiliations:** ^1^ Australian Centre for Ancient DNA, School of Biological Sciences, University of Adelaide, Adelaide, SA, Australia; ^2^ School of Mathematics and Statistics, The University of Melbourne, Parkville, VIC, Australia; ^3^ School of Culture History and Language, The Australian National University, Canberra, ACT, Australia; ^4^ Centre of Excellence for Australian Biodiversity and Heritage (CABAH), School of Biological Sciences, University of Adelaide, Adelaide, SA, Australia; ^5^ National Centre for Indigenous Genomics, Australian National University, Canberra, ACT, Australia; ^6^ Telethon Kids Institute, Indigenous Genomics Research Group, Adelaide, SA, Australia

**Keywords:** Indigenous peoples of America, Native Americans, immunity, colonization, immunogenetic adaptation, infectious disease, host-pathogen coevolution, Virgin Soil

## Abstract

The introduction of pathogens originating from Eurasia into the Americas during early European contact has been associated with high mortality rates among Indigenous peoples, likely contributing to their historical and precipitous population decline. However, the biological impacts of imported infectious diseases and resulting epidemics, especially in terms of pathogenic effects on the Indigenous immunity, remain poorly understood and highly contentious to this day. Here, we examine multidisciplinary evidence underpinning colonization-related immune genetic change, providing contextualization from anthropological studies, paleomicrobiological evidence of contrasting host-pathogen coevolutionary histories, and the timings of disease emergence. We further summarize current studies examining genetic signals reflecting post-contact Indigenous population bottlenecks, admixture with European and other populations, and the putative effects of natural selection, with a focus on ancient DNA studies and immunity-related findings. Considering current genetic evidence, together with a population genetics theoretical approach, we show that post-contact Indigenous immune adaptation, possibly influenced by selection exerted by introduced pathogens, is highly complex and likely to be affected by multifactorial causes. Disentangling putative adaptive signals from those of genetic drift thus remains a significant challenge, highlighting the need for the implementation of population genetic approaches that model the short time spans and complex demographic histories under consideration. This review adds to current understandings of post-contact immunity evolution in Indigenous peoples of America, with important implications for bettering our understanding of human adaptation in the face of emerging infectious diseases.

## Introduction

During the colonial period of the 15th–20th centuries, European nations conquered the globe and dramatically altered the demographic, social, and cultural landscape of other continents. The negative impacts of this global expansion for Indigenous populations are well-documented and persist to this day (Coates, 2004; Glenn, 2015; Paradies, 2016). Most notably in the Americas, Indigenous communities suffered considerable cultural upheaval and societal collapse. Upon European contact and the resulting killings, poor social conditions, and epidemics, Native Americans underwent a severe decline in population, with depopulation estimates falling between 75% and 95% (Dobyns, 1966; Livi-Bacci, 2006). While diseases introduced by European colonists are frequently held accountable as one of the leading causes of depopulation, the current understanding of the biological consequences of European contact remains very limited. In particular, the identification of the causal infectious agents, the spatial and temporal scale of potential epidemics, and the proportion to which different pathogens may have contributed to overall indigenous mortality remain largely unknown (Lovell, 1992; Larsen, 1994; Ramenofsky, 2003).

Understanding the dynamics of large-scale, pathogen-related depopulation events is of great importance, especially considering that infectious diseases are among the strongest selective pressures affecting the evolution of the human genome (Barreiro and Quintana-Murci, 2010; Enard et al., 2014, 2016; Karlsson et al., 2014; Quintana-Murci, 2019). Many infectious agents carried by the Europeans into the Americas have no known prior coevolutionary history with the immune system of Indigenous peoples of the Americas and are widely presumed to have contributed to the unprecedented levels of disease and death among them (Merbs, 1992).

Here, we review the genetic literature investigating the extent to which colonialism has impacted the genomes of Indigenous peoples of the Americas, with an emphasis on the role of infectious disease pathogens and their coevolution with human populations. We specifically focus on the Americas due to the estimated large-scale impacts of colonization on native populations, the greater availability of historical records, and the relatively larger representation of Indigenous Americans in genetic studies than other underrepresented Indigenous peoples.

### Europeans share an extensive coevolutionary history with zoonotic pathogens

European ancestors began to transition from a hunter–gatherer to an agricultural lifestyle around 13–8 ka ago, when animal husbandry practices became more widespread across Eurasia (Diamond and Bellwood, 2003; Guiry et al., 2016; Ethier et al., 2017). The domestication of animals, especially when accompanied by close cohabitation, has been posited as an amplifying step for the spread of zoonotic disease in human populations (Morand et al., 2014; Rahman et al., 2020). Zoonotic pathogens make up the majority of all human-infecting pathogens and are approximately twice as likely to correlate with emerging diseases as pathogens of non-zoonotic origin (Taylor et al., 2001). Some of the most lethal pathogens introduced to Indigenous peoples of the Americas, the so-called “civilization pathogens” (i.e., measles, mumps, tuberculosis, diphtheria, smallpox, and influenza), all share domestic animal reservoirs in western Eurasia (Merbs, 1992; Larsen, 1994; Wolfe et al., 2007). Measles, mumps, and tuberculosis most likely evolved primarily from bovine reservoirs, smallpox is thought to have evolved from horses, diphtheria has etiological agents in most contemporary livestock and pets, and influenza appears to have evolved from avian and swine hosts (Burkovski, 2014; Recht et al., 2020).

Urbanization began in various regions of Europe and Asia during the Late Neolithic to Early Bronze age (∼9–5 ka ago) and is thought to have resulted in regional increases in population density, excess waste, and unclean water in early settlements (Çevik, 2007; Ullinger et al., 2015; Golani and Yannai, 2016; Fernández-Götz, 2018). These dynamics are thought to have facilitated the transfer of pathogens between and within humans and other animals, effectively cultivating an extensive reservoir in which pathogens could evolve (Pearce-Duvet, 2006; Woolhouse and Gaunt, 2007). Several zoonotic pathogens are also hypothesized to have been carried by domesticates traded between countries along the Silk Road, linking together pathogen transfer between Asia and Europe. Along the southern Silk Road route, patterns of several modern *Brucella* strains support continuous expansion, replacement, and spread (Liu Y et al., 2021). It is also hypothesized that *Yersinia Pestis* has been carried along the Silk Road, especially along the northern route, with the timing of its polytomy corresponding to military invasions and population movements across Asia beginning in the 13th century CE (Hymes, 2014). Taken together, the advent of urbanization, potentially lowered levels of hygiene, and higher instances of domesticate movement throughout Eurasia may all have resulted in geographically widespread, multi-host pathogen reservoirs, with potential for molding immunity adaptation in Europeans.

Paleomicrobiological evidence shows that several pathogens appear to share an emergence coinciding with the beginning of agricultural and urbanizing shifts in Europe. Sequences of *Salmonella enterica*, the bacterial cause of enteric (typhoid) fever, were extracted from 6.5-ky-old skeletons of western Eurasian transitional foragers; these ancient *S. enterica* strains cluster with generalist cross-mammalian strains, while modern strains appear to have evolved a specificity for humans in Europe in the last ∼5,000 years (Key et al., 2020). A phylogeny of ancient and modern strains of *Mycobacterium tuberculosis* yields an emergence date of ∼ 2–6 ka ago, again appearing to coincide with the agricultural revolution in Africa (Sabin et al., 2020). *Mycobacterium leprae*, causing leprosy, was most prevalent in Europe around the 12th and 14th centuries CE, declining in 16th-century Europe while simultaneously increasing in other regions of the world (Nerlich and Zink, 2008). Ancient sequences of *M. leprae* have revealed high strain conservation, low mutation rate, and no discernible reduction in virulence compared to modern strains, observed across Europe. From this, it has been hypothesized that the 16th-century decline in European leprosy cases may be explained by selective changes in European host immunity, possibly allowing more recent populations to better combat the detrimental effects of *M. leprae* infection (Schuenemann et al., 2018; Pfrengle et al., 2021). However, the specific mechanisms through which selection putatively acted remain unknown, with the perceived reduction in European incidence/lethality possibly caused by non-selective, random evolutionary forces.

Even considering that European populations might have adapted to some pathogens over their longer times of exposure, there are several examples of pathogens that have continuously infected these populations with high fatality rates. Measles and smallpox are both estimated to have first begun infecting Europeans sometime during the 6th century BCE (Düx et al., 2020; Mühlemann et al., 2020). Despite this early emergence date and several thousand years of coevolution with humans, smallpox and measles continued to devastate Europe, with an estimated mortality rate of 30% until the advent of vaccines and 19th-century eradication programs (Fenner, 1984; Okwo-Bele and Cherian, 2011). Europe was also ravaged by three major plague waves between the 6th and 20th centuries BCE, caused by the bacterium *Yersinia pestis*, collectively killing hundreds of millions of Europeans (Perry and Fetherston, 1997; Wagner et al., 2014). Several studies indicate *Y. pestis* was already infecting ancient Europeans from at least 5.1 ka ago, with evidence of a long, extensive coevolution with this pathogen for populations in both Europe and Asia (Rasmussen et al., 2015; Andrades Valtueña et al., 2017; Rascovan et al., 2019 (Spyrou et al., 2016); Spyrou et al., 2019; Susat et al., 2021; Andrades Valtueña et al., 2022). These repeated outbreaks in Eurasia throughout the last 2,000 years demonstrate that immune adaptation is a complex process, and when it does occur, adaptation likely represents a long-lasting arms race between humans and pathogens, as proposed in the Red Queen Hypothesis (Van Valen, 1973; Siddle and Quintana-Murci, 2014). Accordingly, the continuing high mortality rates in European populations, observed until the development of suitable vaccines, highlight the challenges of human immune adaptation to these infectious agents. Although still requiring more empirical evidence to be ascertained, it is generally believed that pathogens with high virulence also tend to have high infectivity, possibly resulting in the spread and replacement of a continuously higher number of pathogenic strains over time, to which humans might adapt to (Alizon et al., 2009; Froissart et al., 2010).

### Host–pathogen coevolution dynamics may have differed in the Americas

In contrast to Europe, agricultural development and animal domestication took on a very different form in the Americas. Archaeological evidence suggests that the transition to farming practices evolved regionally and fluidly, with some populations alternating between hunting–gathering and farming through time (Dillehay, 2011). Camelids and guinea pigs were domesticated around 6–8 ka ago and 11–13 ka ago, respectively, though there were no known major disease-causing zoonotic pathogens that may have been transmitted humans from any of these species (Lord et al., 2020; Diaz-Maroto et al., 2021). Furthermore, in many population-dense regions across the Americas, urban structures of Indigenous people were highly organized and included well-developed water storage and distribution systems, possibly aiding in better sanitation and reducing microbial spread (Patterson and Runge, 2002). This may further explain why there appear to have only been a limited number of pre-contact epidemics in the Americas. Reports exist of a hemorrhagic fever epidemic, locally known as *cocolitzi*, caused by an endemic strain of *Salmonella enterica*, at the time of contact in Mexico (Acuna-Soto et al., 2002; Vågene et al., 2018). This bacterium is likely to have caused other epidemics in the history of Indigenous peoples in this region, providing one of the few known instances of pre-European human–pathogen coevolution in the Americas. There is some evidence that the parasite *Trypanosoma cruzi*, causing the endemic Chagas disease, may also be coevolving with Indigenous populations, as admixed individuals carrying alleles at high frequency in Indigenous populations at the human leukocyte antigen (HLA) locus are positively correlated with lower susceptibility to the disease (Casares-Marfil et al., 2021). The only other known endemic diseases which held any potential for causing large-scale mortalities are tuberculosis, treponematosis and, possibly, syphilis, the origins of which remain controversial to this day (Beale and Lukehart, 2020). Of these, only tuberculosis has a putative zoonotic origin in seals, with which there would have been little opportunity for an extensive human–animal pathogen reservoir (Bos et al., 2014; Beale and Lukehart, 2020). While not associated with a high mortality rate, endemic subpopulations of *Helicobacter Pylori*, causing gastrointestinal diseases, are found within Peruvian communities, with overall strain replacement by European subpopulations occurring. A novel subpopulation appears to have emerged among admixed individuals from Lima, with these strains possibly carrying overall higher virulence (Gutiérrez-Escobar et al., 2020).

Although the contrasting histories between Indigenous peoples of the Americas and Europeans indeed support that these two populations underwent very different coevolutionary histories with pathogens for the past ∼23 k years, a generalist view of the relationship between agriculture, urbanization, pathogen evolution, and host adaptive potential can hardly be applied to all host–pathogen interactions throughout the course of human evolution. It is near impossible to determine to what extent Indigenous Americans’ differing lifestyle to that of Europeans would have contributed to their adaptation (or lack thereof) to zoonotic pathogens in general. Furthermore, Indigenous groups have high regional diversity, both genetically and culturally, whereby coevolutionary dynamics and overall pathogen landscapes were certainly very regional. Hence, local populations likely experienced different strains with varying virulence and epidemic potential, developing specific immunogenetic responses to pathogens. At present, our understanding of regional, pre-contact epidemics is hampered by significant under-sampling, particularly of ancient individuals. As also illustrated above, the apparent lack of immune adaptation in contemporary European populations despite extensive pathogen interactions since the Neolithic further complicates the expectations. Hence, it is difficult to predict which pathogens Indigenous populations may have adapted to and whether the lack of known pathogens in their history is due to a lack of records and understudying, or a true representation of the historical pathogenic landscape. Despite this caveat, it can be broadly stated that “civilization pathogens” introduced during colonial times have had disproportionate effects on Indigenous populations across the world (Bramley et al., 2004; Jones, 2006; Penman et al., 2017). At the same time, in the Americas, endemic, non-zoonotic pathogens may have imposed selective pressures that shaped Indigenous immunity allele frequencies. At present, this idea remains a working hypothesis as it has not been possible to definitively identify any specific genetic changes in zoonotic pathogens that universally result in increased virulence or transmissibility nor determine private immunity variation in either European or Indigenous populations that confer them with better adaptation to the respective pathogens they have long-standing relationships with. The extent to which differing evolutionary histories and contrasting pathogen landscapes contributed to Indigenous depopulation in the Americas thus includes many avenues yet to be explored, and some aspects may never be fully answered.

### Paradigms explaining Indigenous depopulation during the European invasion of the Americas

While the spread of Europeans across the Americas was a highly regional process and the mode of post-contact depopulation varied extensively between localities, there was an overall trend of severity and swiftness in Indigenous population decline across the American continents (Ramenofsky, 2003; Jones and DeWitte, 2012). This has led anthropologists to formulate two opposing hypotheses for the main depopulation cause. The most widely accepted hypothesis embraces the idea of differing coevolutionary histories between European and Indigenous populations and assumes that Indigenous Americans carried an “innate susceptibility” to colonial-introduced pathogens. This is referred to as the “Virgin Soil” hypothesis (Crosby, 1976; Thornton, 2005). Eyewitness accounts and historic descriptions of Indigenous populations ravaged by various epidemics are the primary sources of evidence for this hypothesis, although this must be contextualized by methodological uncertainty as diseases were diagnosed by symptoms, and infectious disease pathology was not well-characterized at that time (Spyrou et al., 2019). An alternative hypothesis, referred to as the “Black Legend hypothesis,” explains Indigenous depopulation as a function of interconnected sociological causes, in which disease played a role but was not the sole primary driver (Whitaker and Hanke, 1936; Lovell, 1992; Livi-Bacci, 2006). Sociological factors include poor sanitation, loss of infrastructure, birth rate decline, wars, killings, and translocation of people and famine, as caused by colonial effects of that time (Keen, 1969). Studies have noted that the infectivity and spread rate of the smallpox virus appear to mismatch estimated post-contact depopulation rates, especially considering that the time taken for the pathogen to reach negligible levels of host infectivity is shorter than the transatlantic sailing time. This led scholars to question exactly as to what extent a role was played by infectious diseases in very early post-contact depopulation and whether this may have been more attributed to warfare and killings (Maccallum and Mcdonald, 1957; Wolff and Croon, 1968; Keen, 1969; Crosby, 1976). In certain regions, the “Black Legend” hypothesis cannot be applied on a local scale as there are many strongly supported examples of infectious disease exerting a disproportionate impact upon Indigenous populations. Among these is the case of the Tsimshian population, who suffered a post-contact smallpox outbreak with a death rate of 70% in the Alaskan Haida (Boyd, 1999; Hays, 2005). In the case of early colonial Mexico, although there are no exact numbers describing the depopulation impact of infectious diseases, the devastating consequences of epidemics have been well-described (Dobyns, 1993; McCaa, 1995). Aside from smallpox, influenza, and measles, outbreaks are also thought to have contributed to high mortality in certain regions. Up to 22% of Native Alaskans at St Lawrence Island are thought to have died due to influenza infection (a regional mortality rate of 75%), and up to 50% of deaths were caused by a measles epidemic among the Northern Plains Indigenous groups (Jones and DeWitte, 2012). It is not clear whether these instances were representative of the overall depopulation across the Americas or whether they signified isolated events, with more of the population decline explained by under-recorded sociological causes.

The extent to which these two hypotheses best describe the overall mode of Indigenous depopulation across the entire Americas thus remains a matter of intense debate to this day. Controversial but outspoken scholars have claimed that the true extent of sociologically driven demise, under colonial rule, was underestimated due to politically biased colonial narratives, outrightly rejecting the Virgin Soil hypothesis (Lovell, 1992). Inversely, scholars have also argued that early advocates of Indigenous’ human rights may have minimized reports of infectious disease to highlight the atrocities being suffered under the conquerors’ rule (Joralemon, 1982). Depopulation estimates also rely on estimates of pre-contact census size, which vary extensively from 10 to 120 million for the Americas, with the most recent and perhaps widely accepted inferences settling on around 75–100 million (Thornton, 1987; Livi-Bacci, 2006; Smith, 2017). From an anthropological and archaeological perspective, the high discordance pertaining to Indigenous depopulation in the Americas and the poorly understood contribution of infectious disease highlight the need for the implementation of novel approaches. Accordingly, genetic studies may help provide a clearer picture of the effects of colonial processes in these populations.

### Genetic studies investigating the demographic effects of colonization

Our understanding of human history has been revolutionized by improvements in DNA sequencing technologies and the ability to extract ancient DNA from archaeological contexts. These advancements have allowed genetic-based modeling of major demographic events, from tracing the early peopling of the Americas to detecting fine-scale genetic imprints of colonization-linked demographic movements, admixture events, and selection.

Genetic evidence suggests that anatomically modern humans dispersed out of Africa 50–90 ka ago, with successive population bottlenecks decreasing regional genomic diversity as non-African populations expanded into the rest of the world (Mallick et al., 2016; Nielsen et al., 2017; Liu Z et al., 2021). Current models posit that one of these lineages formed a small founding population in the region connecting the Asian and American continents, where these individuals presumably remained isolated for thousands of years, probably due to the extensive ice sheet expansion during the Last Glacial Maximum (LGM) (Tamm et al., 2007). At the end of the LGM around 18–15 ka ago, the descendants of this small founding group diverged into an Ancestral Native American (ANA) lineage (thereby becoming distinct from other groups such as Paleo-Eskimos). This in turn facilitated the formation of Northern Native American and Southern Native American branches. Populations with either of these ancestries rapidly spread across both American continents, reaching the southernmost regions of South America ∼15 ka (Moreno-Mayar et al., 2018a; Flegontov et al., 2019; Roca-Rada et al., 2021). Studies using genetic data from past and present-day Indigenous American populations support the scenario of a very small founding population, extended population isolation, serial founder effects, as well as rapid dispersal during the peopling of the Americas (Llamas et al., 2016; Potter et al., 2018; Willerslev and Meltzer, 2021). There are several implications of this demographic history in terms of differential potential in host–pathogen response between Indigenous American and European populations. First, the successive population bottlenecks and founder effects resulted in an overall lower effective population size that may have reduced the efficacy of selection-driven allele frequency change. Second, the long-term isolation from other human populations implies that any immunogenetic selection taking place in Indigenous peoples of the Americas would most likely have occurred with specificity for the local pathogenic landscape, a hypothesis aligning with the Virgin Soil premise. However, these hypotheses must be taken in the context of focusing on a very broad overview of Indigenous genetic diversity as current evidence suggests that the genomic landscape of Indigenous populations throughout the Americas is far from homogenous: 1) some populations in South America and Central America exhibit differential relatedness to North Americans, carrying ancestry from the California-Channel Islands (Scheib et al., 2018; Gnecchi-Ruscone et al., 2019; Nakatsuka et al., 2020); 2) the population genetic structure in Indigenous populations of Central America and the Caribbean has been shaped by back migration of South American ancestors since the initial peopling phase (Gravel et al., 2013; Schroeder et al., 2018; Nägele et al., 2020; Capodiferro et al., 2021; Kennett et al., 2022); and 3) some South American Indigenous populations along the Pacific coast and in the Amazonian basin carry an excess of allele sharing with Australasians (Raghavan et al., 2015; Skoglund et al., 2015; Moreno-Mayar et al., 2018b; Posth et al., 2018; Castro E Silva et al., 2021). While some populations, such as the Surui, underwent extensive genetic isolation (Gnecchi-Ruscone et al., 2019), their reduced effective population size and genetic isolation have likely not carried negative implications for pathogen response as the levels of genetic diversity do not correlate with adaptive potential (Teixeira and Huber, 2021). These examples illustrate how Indigenous demographic history must be considered when studying pathogen adaptation in the Americas.

Upon contact with Europeans, the colonial-linked depopulation of Indigenous populations was impactful enough to be detected genetically across multiple regions, especially when using data from ancient individuals. From a time-series of mitochondrial data, ancient lineages spanning back to 8.5 ka ago were absent from known contemporary datasets, although these findings were limited by the small geographical overlap of ancient and present-day datasets. Demographic modeling of these lineages revealed a population bottleneck coinciding with the time of colonization (Llamas et al., 2016). A dataset of two hundred ancient and contemporary mitochondrial genomes, with all major North American lineages represented, also revealed a sharp decrease in overall diversity and a reduction in female effective population size of approximately 50%, coinciding with European arrival around 500 years ago. The broad range of individuals included in this dataset, combined with the severity of the modeled contraction, provides evidence that depopulation was not especially localized and affected Indigenous populations in a widespread fashion throughout the continent (O’Fallon and Fehren-Schmitz, 2011). When studying the nuclear genome, the analysis of exomes from 50 ancient and present-day Native American individuals showed genetic evidence for a population bottleneck ∼175 years ago in North America. This timing coincides with the arrival of several waves of documented colonial-introduced epidemics, including smallpox (Lindo et al., 2016). From modern data only, Indigenous depopulation has been modeled using genetics across various regions of both American continents (Gravel et al., 2013; Browning et al., 2018). Ancestry-specific identity-by-descent methods predicted that the ancestors of present-day Puerto Ricans underwent a reduction in effective population size to less than 100 individuals at the time of European arrival. Large-scale population (and therefore diversity) declines were also inferred in other regions among the Southern Chilean Huilliche–Pehuenche (an estimated decline of 96%), the Mexican Mixe (94%), and the Tsimshian (57%) around the same time (Gravel et al., 2013; Lindo et al., 2016; Browning et al., 2018; Lindo et al., 2018). The variability of these estimates reflects the differing effects of colonialism on Indigenous groups in different regions, with varying magnitude and impact across the American continents.

In addition to modeling colonial-linked depopulation, it has been possible to trace post-contact admixture into modern Indigenous American populations to an unprecedented fine-scale resolution. In Latin Americans, population structure reflects the geographical locations of contemporary Indigenous populations, together with proportions of admixture from South/East Mediterranean, African (from the slave trade), Sephardic (from the clandestine migrations of Christian Jews), and East Asian ancestries. In Brazil, Latin Americans showed highest genetic affinity to Portugal and West-Spanish ancestry, while West/Central American countries showed greater Central/South-Spanish ancestry, in keeping with records of conquests carried out by Spain and Portugal at the time (Chacón-Duque et al., 2018). These regional-specific admixture proportions and inferred gene flow timings coincide with documented historical migrations to the Americas. On an even finer scale, methods using ancestral tract lengths determined a multiple pulse migration model, whereby, after initial European contact, there were additional pulses of European migration between nine and three generations ago, as well as another intermediate pulse of African slave trade migration (Homburger et al., 2015). Similar estimates of post-contact admixture proportions, admixture timings, and sex bias in the Americas have been carried out in a suite of studies using contemporary individuals and show high concordance with historical records, reflecting the diverse and extensive population movements mediated under various European conquerors of the time (Montinaro et al., 2015; Adhikari et al., 2016; Harris et al., 2018; Barbieri et al., 2019; Ongaro et al., 2019). Genetic studies have also successfully reconstructed fine-scale population genetic history both pre- and post-contact in Latin American populations from the Caribbean, Mexico, and Brazil, tracing ancestries from both Indigenous populations and other worldwide regions (Gravel et al., 2013; Moreno-Estrada et al., 2013; Moreno-Estrada et al., 2014; Mas-Sandoval et al., 2019). These insights are important for understanding population structure and demography in the Americas. They also pave the way for better contextualizing and detecting putative selection acting upon both admixed and non-admixed alleles in Indigenous populations.

### Genetic studies investigating European and African admixed alleles under selection in Indigenous populations

From contemporary data, there are several instances of contact-related selection appearing to act on the genomes of Indigenous populations of America. HLA genes are among the most vital loci to a strong immune system as they code for molecules which present antigen peptides on the surface of cells, which can then be recognized by T-cells and trigger a downstream inflammatory response, including the elimination of infected cells (Choo, 2007). The recognition of pathogenic peptides by HLA is thus thought to be necessary (though not sufficient) for immune defense. Previous studies investigating admixed Latin American populations in Colombia, Mexico, Peru, and Puerto Rico suggested that selection may have acted upon admixed alleles from Africa, particularly within the HLA region and in pathways involved in inflammation, such as the innate and adaptive immune response (Norris et al., 2020). Another study on five Latin American cohorts showed that HLA alleles under putative selection carry an overrepresentation of African ancestry (Ongaro et al., 2021; Mendoza-Revilla et al., 2022). This study used deviations from neutrality in the expected allele frequency in admixed populations, given known allele frequencies in the source populations, to allow detection of selected regions despite high levels of admixture. This approach showed that 29 of 47 candidate regions under putative selection (notably related to immunity and infection) in admixed Mexicans originate from non-Indigenous ancestry (British, Han Chinese, or African ancestries), reflecting the potential role of admixed alleles in adaptation. In yet another study, modern European-admixed Chilean populations appeared to show selection affecting regulatory elements and long non-coding RNAs with important functions involved in innate immunity. This was determined by examining regions of the genome with higher-than-expected European ancestry compared to genome-wide estimates (Vicuña et al., 2020).

So far, only a handful of studies have focused on detecting post-contact immunogenetic selection signals using ancient DNA data. An analysis of 50 ancient and modern exomes from Canadian First Nation peoples led to the identification of positive selection signals in the *HLA-DQA1* gene, with several alleles close to fixation, including a polymorphism in the 5’ UTR that suggests selection targeted the regulatory activity of the gene. However, these results were not reproduced when analyzing modern Tsimshian individuals through multiple models of different forms of selection (Lindo et al., 2016). Interestingly, most of the *HLA-DQA1* alleles, including both of its nonsynonymous variants, exhibited a sharp decrease in allele frequency in modern individuals, compared to the ancient individuals. This could be related to a selective advantage to the endemic pathogenic landscape predating European arrival, which changed after colonization due to the different selective pressures (Lindo et al., 2016). However, this hypothesis remains speculative as localized sharp changes in allele frequencies are also compatible with models of neutral evolution. In a different study focusing on highland and lowland Andean populations, the genomes of seven ancient individuals were analyzed alongside a panel of contemporary genetic variation. Using a Population Branch Statistic approach, the authors identified a handful of putatively selected immune candidates in post-contact Andean highlanders that may have involved adaptation to colonial pathogens but again are difficult to affirmatively disentangle from genetic drift (Yi et al., 2010; Lindo et al., 2018).

## Discussion

### Towards a holistic approach in detecting immunogenetic selection in post-contact populations

A wealth of genetic evidence suggests that pathogens are strong drivers of selection on immunity genes in humans (Karlsson et al., 2014; Quintana-Murci, 2019). Across human populations, pathogen load has shown an unexpectedly strong correlation with genetic diversity when compared to various other local geographical factors (including diet and climate). Among other studied functional categories, immunity genes showed the strongest hallmarks of local adaptation to high pathogen pressure—even when correcting for demographic history and population structure (Fumagalli et al., 2011). Distinct subclasses of the immune system have also individually been identified as being subject to disparate forms of adaptive evolution (e.g., balancing selection) (Leffler et al., 2013; Key et al., 2014; Teixeira et al., 2015; Bitarello et al., 2018; O’Neill et al., 2020). The innate immune response, functionally defined as the “first line” of immune defense, comprises genes involved in detection and destruction of pathogens and pathogen-infected cells in the early stages of infection (Ezekowitz and Hoffmann, 2002; Deschamps et al., 2016). Much of the innate system is generalist in its response, interacting with the conserved parts of pathogens that are common to many infectious agents (Alberts et al., 2002). Host innate immune genes are highly conserved themselves, showing stronger signatures of purifying selection than genes in other categories across human populations; a handful of innate immunity genes also show evidence of local, regional positive selection in African, Asian, and European populations (Mukherjee et al., 2009; Deschamps et al., 2016). Genes identified as coding for proteins that interact directly with viruses also show higher rates of purifying selection than other gene groups, while at the same time exhibiting strong signals of directional adaptation compared to the rest of the conserved proteome (Enard et al., 2016). These observations further support the idea of immunity genes, especially those immediately involved in the immediate response to and elimination of exogenous material, being finely tuned by (sometimes opposing) selective forces. This is likely due to their crucial, conserved nature in pathogen defense, while also requiring enough plasticity to adapt to local pathogens attempting to evade host immunity functions on smaller spatiotemporal scales (Mukherjee et al., 2009; Deschamps et al., 2016). HLA genes, another vital facet of the immune response, are extremely polymorphic and carry some of the highest diversity in the human genome, affording them the capability of recognizing a large diversity of pathogens. There is evidence that more generalist HLA alleles, which can bind a wide array of pathogenic peptides, tend to be at higher frequencies in geographical locations with a high recorded diversity of human pathogens (Prugnolle et al., 2005; Manczinger et al., 2019). For both the Northern and Southern American continents, modern Indigenous populations also show a higher frequency of HLA alleles that are predicted to bind strongly to viral peptides, as well as lower frequencies of weakly binding alleles (Barquera et al., 2020). In these populations, HLA allele diversity is especially low, as is the case for killer-cell immunoglobulin-like receptors (KIR), involved in recognizing HLA molecules and triggering an inflammatory response. HLA-KIR molecular interactions are also very limited in Indigenous Americans, with most KIR proteins binding to a few very specific HLA molecules (Vargas et al., 2022). Quantifying the amount of HLA/KIR diversity showing similar patterns prior to European contact, or that has changed since, remains yet to be investigated.

Since immunity genes generally demonstrate such high susceptibility to selective forces, there is a possibility that post-contact Indigenous populations experienced similar effects from introduced pathogens, especially if the mode of Indigenous depopulation in the Americas had (at least partially) occurred as premised under the “Virgin Soil” hypothesis. If introduced pathogens were coevolving with European hosts for several thousand years prior to colonization, the novel selection pressure exerted on immunity genes of Indigenous Americans could produce recent, disparate changes in immune allele frequency, as compared to other populations. Under neutrality, there would be no significant differences in the patterns of genetic variation between immune genes and the remainder of the genome that did not already exist prior to contact. A strong deviation in allele frequency trajectories of immune genes, as opposed to genes involved in other functions, may, therefore, indicate that immune genes were exposed to especially high post-contact selective pressures. This highlights the need for ancient genomes to inform the frequency of pre-contact immunity-related alleles and monitor the frequency trajectories of introduced European alleles that could confer a selective advantage. However, even with this approach, there are still many difficulties in disentangling this signal from random genetic drift and other confounding factors generated by demographic processes. In the case of highly admixed, contemporary Indigenous individuals, signals of selection could nonetheless be obscured by the mixing of alleles from different ancestries at various frequencies (Souilmi et al., 2021). Given that pathogens were introduced to the Americas 500 years ago, it is likely that regions of the genome, even under strong selection pressure, would currently be still increasing in frequency and that some selected variants may not even yet exist in Indigenous populations. The recency of colonization may thus mask the detection of selected genomic regions (Schrider and Kern, 2017; Vargas et al., 2022). While recent analyses have identified selection signals possibly linked to colonization by modeling allele frequency deviations considering admixture, the candidate loci exhibit very large selection coefficients (∼0.15–0.2) that enhance statistical power (Zheng and Wiehe, 2019; Mendoza-Revilla et al., 2022). Taking these limitations into account, pathogen-driven selection acting on post-contact immunity genes in Indigenous peoples of the Americas would thus be best identified by population genetic methods that can detect soft selective sweeps, comparisons between ancient and contemporary genomes, comparisons between worldwide populations, and polygenic approaches, wherein selection signals are observed cumulatively across immune gene classes and pathways. In addition, it is crucial to develop and implement theoretical and computational tools that allow effectively modeling the complex demographic history of these populations and accurately account for the effects of genetic drift. This could be achieved with admixture graphs comparing deviations in allele frequencies in specific branches versus neutral expectations (Refoyo-Martínez et al., 2019). These methods can accommodate both modern and ancient data and can more precisely account for complex demographic histories, making them more accurate than current approaches using broad-scale continental data (Rishishwar et al., 2015; Norris et al., 2020; Vicuña et al., 2020; Mendoza-Revilla et al., 2022).

Finally, as is the case for all approaches based on indirect measures, any immune genes suspected to be under contact-linked, pathogen-driven selection require functional validation to elucidate the biological mechanisms at play. These mechanisms could be further complicated by microbiome composition and epigenetic modifications, both of which might play crucial roles in maintaining immune homeostasis, immune regulation, and resistance or adaptation to pathogens (Wu and Wu, 2012; Obata et al., 2015). Several experimental approaches examined ancestry-specific immune responses and revealed that immunity-linked expression quantitative trait loci are highly differentiated between European and African populations (Randolph et al., 2021). Specifically, increased European ancestry was associated with a stronger type I IFN response to influenza and thus was a predictor of reduced viral titers following infection. Using similar approaches, other studies have also uncovered a differential transcriptional response in European and African monocyte-derived cells to pathogenic stimuli, with African ancestry correlating with a stronger inflammatory response (Nédélec et al., 2016; Quach et al., 2016). Such studies are currently needed for Indigenous populations and would be well-strengthened with detailed investigation of the biological mechanisms for each gene driving potential population-specific signals. The investigation of these mechanisms may be aided by comparing selection acting on immune genes in other populations. Indigenous populations around the world underwent long periods of isolation prior to European conquest, most apparent in the Pacific islands and Australia. Again, populations evolved with significantly different domestication practices and pathogenic landscapes to those of Europe, possibly contributing to a higher susceptibility to introduced infectious disease. While colonization is highly multifaceted and immune selection signals would vary depending on the population and temporal occurrence of epidemics, convergent evolution of immunity genes across global Indigenous populations is a very strong possibility and could highlight key genetic players in immune adaptivity. Comparing selection signals from Indigenous peoples of Australia and America would be especially powerful due to their similar histories of isolation, parallel demographic consequences of colonization, and no pre-contact exposure to Eurasian pathogens.

The importance of ameliorating our understanding of the dynamics of Indigenous depopulation in the Americas, especially in the context of infectious disease, cannot be understated. For Indigenous people, the aftermath and detrimental effects of post-contact events are still rife today, as demonstrated by marked health and sociological disparities, and exacerbated by historical bias toward colonial narratives and understudying of Indigenous populations (Gracey and King, 2009; Axelsson et al., 2016). Previous genetic studies, enhanced using ancient DNA, have demonstrated that many aspects of post-contact demographic effects and their imprints on Indigenous American genomes can be detected at a fine-scale resolution. There is still much to be discovered, especially regarding the immunogenetics of Indigenous American populations before exposure to European-borne pathogens, how immune genes have evolved since colonization, and which genes were selected as a result. These insights will inform the processes of human and pathogen coevolution, an area that is especially relevant for managing both Indigenous and non-Indigenous health, as well as safeguarding against future emerging infectious diseases.
